# Patients with Non-Obstructive Coronary Artery Disease Require Strict Control of All Cardiovascular Risk Factors: Results from the Polish Local Population Medical Records

**DOI:** 10.3390/jcm10122704

**Published:** 2021-06-18

**Authors:** Jarosław Hiczkiewicz, Paweł Burchardt, Jan Budzianowski, Konrad Pieszko, Dariusz Hiczkiewicz, Bogdan Musielak, Anna Winnicka-Zielińska, Daria M. Keller, Wojciech Faron, Janusz Rzeźniczak

**Affiliations:** 1 Department of Interventional Cardiology, Collegium Medicum, University of Zielona Góra, 65-046 Zielona Góra, Poland; jhiczkiewicz@uz.zgora.pl (J.H.); kpieszko@uz.zgora.pl (K.P.); dhiczkiewicz@uz.zgora.pl (D.H.); bmusielak@uz.zgora.pl (B.M.); 2 Department of Cardiology, Nowa Sól Multidisciplinary Hospital, 67-100 Nowa Sól, Poland; pburchardt@ump.edu.pl (P.B.); awinnik@tlen.pl (A.W.-Z.); wfaron@poczta.onet.pl (W.F.); 3 Department of Hypertension, Angiology, and Internal Medicine, Poznan University of Medical Sciences, 61-848 Poznań, Poland; 4 Department of Cardiology, J. Strus Hospital, 61-285 Poznań, Poland; syngo@wp.pl; 5 1st Department of Cardiology, Poznan University of Medical Sciences, 61-848 Poznań, Poland; daria.keller@skpp.edu.pl

**Keywords:** subsequent percutaneous coronary intervention, no obstructive coronary artery disease, coronary angiography

## Abstract

The aim of the project was to compare patients treated with percutaneous transluminal coronary angioplasty (PTCA), who also had undergone PTCA in the past, with a group of people who had had no angiographic stenosis in the lumen of the coronary arteries in the past, and who also required PTCA during index hospitalization. The secondary aim was to compare the obtained data with the characteristics of a group of people who had undergone angiography twice and for whom no significant stenosis had been found in their coronary arteries. The study used registry data concerning 3085 people who had undergone at least two invasive procedures. Acute coronary syndrome (ACS) was significantly more often observed (Non-ST-segment elevation myocardial infarction (NSTEMI) OR 2.76 [1.91–3.99] and ST-segment elevation myocardial infarction (STEMI) OR 2.35 [1.85–2.99]) in patients with no significant coronary stenosis in the past (who required coronary angioplasty at the time of the study), compared to patients who had already had PTCA. They also demonstrated more frequent occurrence of ‘multivessel disease’. This was probably most likely caused by inadequate control of cardiovascular risk factors, as determined by higher total cholesterol levels ([mg/dL] 193.7 ± 44.4 vs. 178.2 ± 43.7) and LDL (123.4 ± 36.2 vs. 117.7 ± 36.2). On the other hand, patients in whom no significant stenosis was found in two consecutive angiographies were more likely to be burdened with chronic obstructive pulmonary disease, atrial fibrillation and chronic kidney disease.

## 1. Introduction

In the 20th century, significant medical advances improved the treatment methods of coronary artery disease (CAD), which is the most common cause of death. The etiopathogenesis of CAD, which determines the main risk factors, was successfully defined. Additionally, the possibility of diagnostic visualization of coronary arteries was achieved, and various techniques were finally developed to perform revasularization. Numerous literature reports confirm that the performance of percutaneous transluminal coronary angioplasty (PTCA) or the diagnosis of atherosclerosis also determines further optimal medical treatment (OMT), the effectiveness of which depends on the compliance with medical recommendations and the use of pharmacotherapy [1]. The more we know about the disease itself and the methods of treating it, the more narrow areas of investigation or intricate not fully studied issues appear. Indeed, they all pose many scientific questions and simply arouse curiosity. One of them is, for example, the clinical characteristics of patients who have been qualified to undergo narrowed vessel angioplasty, and who had angiography performed in the past with no significant stenosis found. This is why, in this study, the patients who underwent angioplasty of a narrowed coronary artery during index hospitalization were split into two groups: the patients in which PTCA was also performed in the past and the ones which had no significant stenoses in the lumen of coronary arteries in the past. The two groups were compared with each other.

It was also scientifically intriguing to compare the clinical characteristics of patients who had undergone coronary angiographies several times (with no significant narrowing lesions in the lumen of the arteries found) with the patients who had undergone coronary angioplasty. This was the secondary goal of the project.

## 2. Materials and Methods

### 2.1. Study Population

The study included recorded data from 3085 people (2445 included in the coronary artery disease group + 640 subjects in the control group) who underwent hospitalization at the Cardiology Department, Multidisciplinary Hospital Nowa Sól, between 1 January 2009 and 15 May 2015. The study was retrospective, carried out in accordance with the principles of the Helsinki Declaration and did not require separate approval of the bioethics committee.

The study was carried out on the subjects who had undergone at least two invasive procedures, while the data collected during the second procedure constituted the analyzed ‘output data’. Of the subjects, 2445 patients underwent angioplasty of all significantly narrowed coronary arteries during index hospitalization. Stenosis was measured by quantitative coronary angiography (QCA), and those arteries with a diameter reduced by more than 70% (according to current standards) were regarded as significant. In questionable cases, fractional flow reserve (FFR) was undertaken according to the European Society of Cardiology (ESC) guidelines; however, these were sporadic. Regrettably, it was not possible to perform FFR in all the cases given the limited availability of the procedure in Poland in 2009–2015—the time when the project was carried out.

The patients from the CAD groups were divided into the following subgroups with respect to the history of coronary angioplasty and myocardial infarction:

Subgroup A included 1328 patients who underwent coronary angiography in the past (no significant lesions were found there) and PTCA during index hospitalization.

Subgroup B comprised 434 patients. Previously, they had PTCA performed given a negative myocardial infarction (MI) history. Another PTCA was performed during index hospitalization.

Subgroup C included 683 patients. Previously, they had PTCA for MI performed and another PTCA was carried out during index hospitalization.

The remaining 640 patients made up the control group (CG), which was described in our previous project [2]. Previously, no significant coronary artery stenoses during angiography were found in the past, nor during index hospitalization.

The data obtained from patients during index hospitalization were analyzed in light of specific anatomical and morphological features found in the coronary angiograms, the type of techniques used during interventional procedures and the presence of particular clinical features.

The mean observation time between procedures in subgroup A was 1281 days, 230 days in subgroup B and 944 days in subgroup C.

The exclusion criteria in the study were as follows:

- history of coronary artery bypass graft (CABG) or qualification for this procedure during the observation period;
- significant stenosis of the left main coronary artery;
- coexisting heart defects;
- concomitant severe NYHA III/IV heart failure.

**Figure 1:**
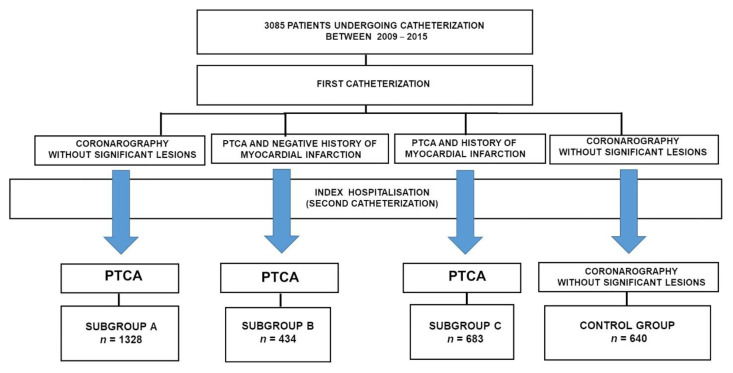
Flow chart of the study. PTCA—Percutaneous transluminal coronary angioplasty.

The study flow chart is presented in Figure 1.

### 2.2. Laboratory Assessments

Although blood was collected at each intervention, laboratory tests relied on the material obtained during the second angiography. In the analyses, peripheral blood count was measured with CELL-DYN Ruby (Abbott Diagnostics, Santa Clara CA, USA). TnT was measured with a Cobas 6000 device (Roche Diagnostics GmbH, Mannheim, Germany) with a cut-off value of 14 pg/L. Creatinine, total cholesterol, triglycerides and high-density lipoproteins were analyzed by means of a photometric test (Roche Diagnostics GmbH, Mannheim, Germany).

### 2.3. Statistical Analysis

The analysis focused on interval and nominal scale data. Interval data, i.e., laboratory results, were presented as mean and standard deviation. Comparisons were made between more than two groups; one-way analysis of variance (ANOVA) was used together with Tukey’s post hoc test or the Shapiro–Wilk test as well as Levene’s test; the Kruskal–Wallis test and post hoc Dunn’s test were employed as an alternative. Subsequently, one-dimensional logistic regression models were applied, followed by multi-dimensional logistic regression models. The results obtained were presented as an odds ratio (OR) with a 95% confidence interval (CI). The survival analysis results are presented graphically as Kaplan–Meier curves. Statistical analysis was performed using STATA15.1 software (College Station, TX, USA). All tests were analyzed at alpha significance level = 0.05.

The study was retrospective, carried out in accordance with the principles of the Helsinki Declaration, and did not require separate consent of the bioethics committee.

The data concerning a 60-month survival period were collected prospectively using updated electronic data from the national health care system.

## 3. Results

### 3.1. Characteristics of the Studied Groups and Differences between the CAD Group and the CG

The characteristics of the coronary artery group and the control group, together with the differences between the groups, are presented in Table 1 and Table 2. Although the coronary artery disease (CAD) group and the control group (CG) were described previously in [2], the context of the description was completely different. 

The characteristics are provided in Table 1 and Table 2.

### 3.2. Characteristics of Patients without Previous Cardiovascular Intervention vs. Patients with History of PTCA and MI

Subgroup A: 1328 patients without MI who had undergone coronary angiography and who underwent PTCA during index hospitalization.

Subgroup B: 434 patients with a history of PTCA and negative history of myocardial infarction. Another PTCA was performed during index hospitalization.

Subgroup C: 683 patients with past MI and PTCA, who had their second PTCA performed during index hospitalization.

In comparison to subgroup A, arterial hypertension was observed significantly more often in subgroups B and C. A similar percentage of diabetes was observed in A, B and C as well as in the CG, except for insulin-dependent diabetes. Heart failure (NYHA I and II) was slightly more frequent in C (patients after MI), i.e., 16.0% vs. 12.3% in A and 8.7% in B (*p* < 0.002). In the CG, incidence of heart failure was 15.0%. With respect to COPD, A, B and C did not differ significantly and the highest risk of its occurrence was observed in the CG (Table 3 and Table 4). Additionally, A, B and C demonstrated no significant differences in terms of history of stroke; the percentages were, respectively 4.7%, 3.2% and 5.2%.

The percentage of cigarette smokers was clearly lower in B and C compared to A.

Further characteristics are provided in Table 3 and Table 4.

### 3.3. Multivariate Regression Results

**Figure 2:**
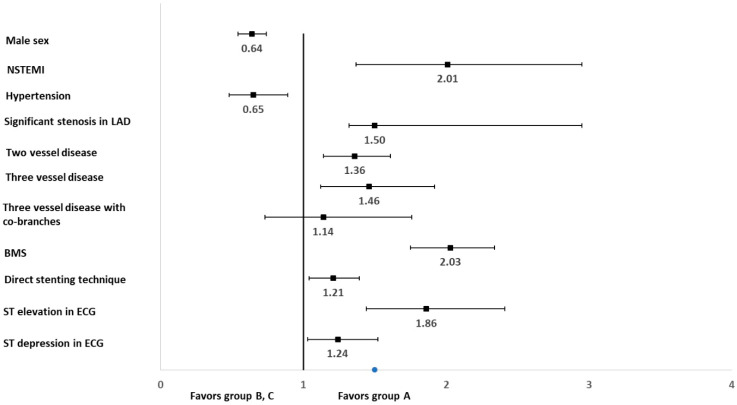
The results of multivariate regression analysis carried out with the parameters which significantly differentiated groups A vs. B and C in the univariate analysis. Abbreviations: NSTEMI—Non-ST-segment elevation myocardial infarction; LAD—left artery descending; BMS—bare metal stent; ECG—electrocardiography.

The results of multivariate regression analysis carried out with the parameters which significantly differentiated groups A vs. B and C in the univariate analysis are shown in Figure 2.

### 3.4. Analysis of Baseline Coronary Angiography (Second Invasive Procedure)

The risk of significant left anterior descending (LAD) narrowing in coronary angiography performed during index hospitalization was lower by half in B and C when compared to A, with ORs at 0.5 and 0.63, respectively. The incidence of significantly narrowed left circumplex (LCx) was almost identical in the three groups and amounted to 28–29%. The risk of significantly constricted right coronary artery (RCA) was 38% and 13% lower in B and C compared to A. The respective estimated ORs were 0.62 and 0.87.

### 3.5. Analysis of the Initial Electrocardiography Record (Second Invasive Procedure)

Atrial fibrillation was significantly less common in all the patients with CAD compared to CG. ST segment elevation (regardless of location) was several-fold less frequent in B and C vs. A. Similarly, ST segment depression occurred considerably less frequently in B and C vs. A. Negative T wave was observed significantly more often in CG and C (patients after myocardial infarction) compared to B and A. Pathological Q wave was most often observed in group C (19.3%), with lower percentages in B (4.6%) and A (6.3%), resulting in statistically significant differences (*p* < 0.001).

In the CG, heart rate was significantly higher compared to patients from subgroups A, B and C: 82/min vs. 73/min, 69/min, 71/min, respectively (*p* < 0.001).

### 3.6. Analysis of Data from Initial Echocardiography (Second Invasive Procedure)

Interpretable echocardiographic data were obtained from 793 patients, i.e., 23.3% of the entire population. Ejection fraction (EF) in A, B and C was 45.8%, 44.9% and 42.1%, respectively (*p* < 0.041). In the CG, EF was 54.4% (*n* = 180). Contractility abnormalities were assessed in two anterolateral anatomical regions with an interventricular septum and a posterolateral septum. Hypokinesis and/or akinesis on the anterolateral and posterolateral walls were detected significantly more often in C vs. A and B, *p* < 0.05.

### 3.7. Assessment of Total Mortality

**Figure 3:**
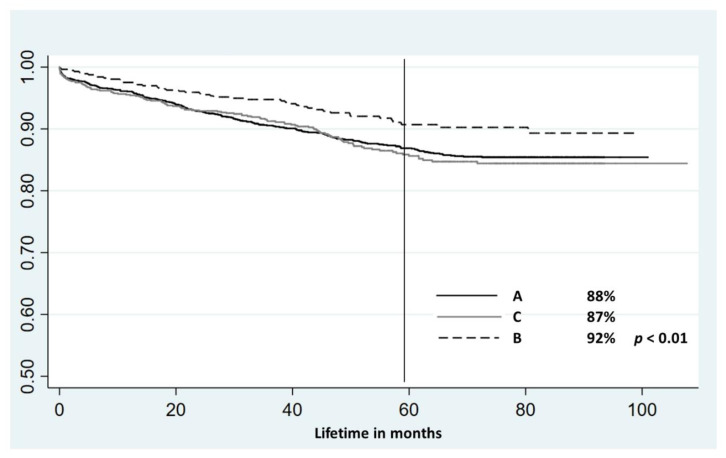
Survival curves for subgroups: 60-month survival in respective subgroups. Legend: Subgroup A—patients without MI who had undergone coronary angiography in the past and who underwent PTCA during the second hospitalization. Subgroup B—patients who had a history of PTCA but did not have a history of myocardial infarction (MI). During the index hospitalization, they had a second PTCA. Subgroup C—patients with past MI and PTCA, who had their second PTCA performed during index hospitalization.

During the follow-up period, the absolute number of deaths was significantly greater in A (13.5%) and C (13.0%) compared to B (8.1%), with *p* < 0.0026. Survival calculated from Kaplan–Meier curves following 60 months of observation in subgroups A, B and C was 88%, 92% and 87%, respectively (*p* < 0.01) (Figure 3).

## 4. Discussion

Every year, about 4 million coronary angiography procedures are performed in Europe and the USA, but in as many as 50% of subjects, no significant stenosis is found in the lumen of the coronary arteries [3]. The cause of their clinical symptoms in those cases is most likely coronary microcirculation disease and/or vasospastic angina [3]. Patients with stable CAD are classified into a generally defined non-obstructive coronary artery disease (INOCA) group, distinguished in a manner similar to those presenting myocardial infarction with non-obstructive coronary arteries (MINOCAs), who are admitted to hospitals as acute coronary syndrome (ACS) cases. There are more and more clinical observations dedicated to these patients [3,4,5,6,7]. It is also acknowledged that in patients in whom coronary angiography showed no significant changes, symptoms of angina are reduced by nearly a half [8,9]. Regrettably, the pertinent literature offers no accurate data on the implementation of the prescribed pharmacological treatment (adherence and compliance) in these particular population groups of patients, while data on the their subsequent fate are considerably limited [10,11].

One must also remember that insignificant hemodynamic lesions in the coronary lumen arteries may also be the cause of future ACS or, after several years of stable disease progression, may cause symptoms of ischemia requiring further intervention. Our results seem to confirm this hypothesis, which is a spectacular result of this project.

Our study included the patients who underwent angioplasty for the first time but, importantly, no significant narrowing lesions were found in the coronary angiography they had had in the past. Compared to the patients with previous PTCA, ACS (Non-ST-segment elevation myocardial infarction (NSTEMI) and ST-segment elevation myocardial infarction (STEMI)) was observed significantly more often in this group; multivessel disease occurred more often as well, while TC and LDL levels were elevated. We may only speculate that adherence to OMT recommendations had been insufficient or completely neglected in those patients, which did not inhibit the natural progress of atherosclerosis and as a result caused it to be more advanced than in the other studied groups. Another aspect seems to confirm this observation, namely the interval between invasive procedures: 1,281 days among the patients from subgroup A, compared to 230 days in B and 844 days in C. As can be seen, the patients whose first angiography showed no significant lesions had another intervention after 3.5 years. The delay in performing the next examination in this group of people seems to contradict the hypothesis of accelerated progression of atherosclerosis, and appears to provide evidence for its natural course.

It may be conjectured that unlike the patients with previous PTCA, the individuals from that particular group would present less advanced atherosclerotic lesions, but the obtained results yet again contradict such a premise. Thus, the group of people who underwent PTCA five times during the follow-up comprised as much as 64% of those who had not demonstrated significant stenosis in the lumen of the coronary arteries during the first angiography. In addition, they also had a higher number of critically altered coronary arteries observed during index hospitalization (unpublished data). Apart from the aforementioned inadequate therapeutic management in those groups, incorrect assessment of atherosclerotic lesions in the coronary arteries during the first angiography may also account for such a result. The phenomenon in question is reported in the literature [12,13,14,15].

In the group of patients with normal angiographic images from the first coronary angiography, critically altered LAD and multivessel disease were observed more often during index hospitalization in comparison to the group of subjects with a history of both PTCA and MI. This is consistent with the few pertinent scientific reports, which observe that among those with insignificant changes in coronary arteries, patients with a diameter narrowed by 20 to 50% are most exposed to CAD progression when compared to people with narrowing lesions below 20% [16,17,18].

Given that our team did not have access to such accurate angiographic data, an analysis of this kind was not performed as part of this project.

Nevertheless, patients with insignificant coronary stenosis or no evidence of atherosclerotic plaques in the vascular bed should be methodically and closely monitored, with a view to maintaining rigorous control of cardiovascular risk factors, as well as ensuring adherence to and compliance with OMT against CAD.

In the present clinical conditions, further invasive diagnostic procedures such as coronary fraction reserve (CFR), FFR and acetylocholine (ACH) tests are also performed in these patients to determine microvascular angina (MVA) or vascular spasm angina (VSA) features [3]. This did not take place as part of our observation study and certainly represents a significant limitation of the project. That particular group should be also diagnosed with other causes of their conditions, as our study demonstrated that atrial fibrillation (AF) or chronic obstructive pulmonary disease (COPD) can also play a role in this respect.

Patients after coronary stent implantation are more likely to take medications as well as to cooperate better with their physicians. Nevertheless, the EuroAspire V study shows that only about 29% achieve therapeutic goals for lipid concentration, approximately 58% achieve proper blood pressure values and only 54% reach the glycated hemoglobin goal [19]. It may be interesting to note that although about 75% of all patients declare full adherence and compliance, the actual failure to comply observed in this group of subjects is responsible for 9% of all cardiovascular events in Europe [19].

Nevertheless, these unsatisfactory data still translate into a better clinical course of coronary artery disease in these patients in comparison to those with angiographically implicit CAD, which indirectly follows from our analysis.

### Limitations of the Study

In this study, the patients with severe heart failure (NYHA III/IV), left main stenosis, extensive atherosclerotic lesions qualified for CABG, concomitant heart defects and primary myocardial diseases were excluded. The criterion of significant lumen narrowing (>70% in diameter) was adopted as a guideline (the analysis did not take into account the fate (observation of survival) of the patients with atherosclerotic lesions in the range of 30–70%, not requiring stenting according to current standards [1,2]). The study was a single-center retrospective analysis and we had no data regarding, e.g., the number of patients with implanted peacemakers.

The population included patients with both ACS and stable coronary artery disease. However, the control group was not homogeneous regarding the standards of ambulatory care, i.e., the frequency of follow-up visits, as well as the type of pharmacotherapy. The group of people with insignificant coronary artery stenosis was referred to general practitioners’ (GP) care. Most of them had their next cardiac catheterization due to acute coronary syndrome.

## 5. Conclusions

In the patients with no significant coronary stenosis in the past, who subsequently required coronary angioplasty during index hospitalization, angiographic advancement of atherosclerosis was greater compared to those who had PTCA in the past (as well as required repeated PTCA during index hospitalization). This may be related to a better control of the disease risk factors and significantly better adherence to treatment among those with angiographically proven CAD. On the other hand, those who did not demonstrate significant stenosis in two consecutive angiographies were more likely to be burdened with COPD, AF and CKD, which could be misconstrued as CAD.

## Figures and Tables

**Table 1:** Characteristics of the studied group.

Parameter	Coronary Artery Disease Group *n* = 2445	Control Group *n* = 640	*p*-Value
Age [years]	64.8 ± 9.8	66.6 ± 8.9	*p* = 0.0005
Male sex [%]	68	61.7	*p* = 0.0081
Hypertension (AH) [%]	92.7	82	*p* = 0.0000
Diabetes (DM) [%]	24	21	*p* = 0.1639
Heart failure by NYHA I/II [%]	10	15	*p* = 0.0013
Chronic kidney disease (CKD) [%]	3	15	*p* < 0.0001
Chronic obstructive pulmonary disease (COPD) [%]	4	12	*p* < 0.0001
Claudication	6	12.8	*p* < 0.0001
Dyslipidaemia [%]	26	40	*p* < 0.0001
Stroke [%]	4.7	2.3	*p* = 0.0214
Smoking [%]	11.6	15	*p* = 0.0081

**Table 2:** Results of one-dimensional logistic regression for CAD vs. CG (where the CAD group was the reference).

Parameter	OR	95% CI
Age during procedure	1.02	[1.01; 1.03]
Male sex	0.73	[0.59; 0.89]
Hypertension	0.36	[0.27; 0.48]
Insulin-dependent DM	0.17	[0.09; 0.31]
CKD	2.83	[2.1; 3.81]
COPD	2.3	[1.67; 3.18]
Intermittent claudication	1.49	[1.09; 2.01 ]
Ischemic stoke	0.48	[0.25; 0.92]
Sinus rhythm	0.45	[0.34; 0.59]
AF	3.02	[2.25; 4.07]
Lack of R progression in ECG	4.37	[3.43; 5.57]

Abbreviations: AF—atrial fibrillation; CKD—chronic kidney disease; COPD—chronic obstructive pulmonary disease; DM—diabetes mellitus; type 2 diabetes; AH—arterial hypertension; ECG—electrocardiography; OR—odds ratio; CI—confidence interval.

**Table 3:** Patient characteristics: history of cardiovascular interventions vs. history of PTCA and myocardial infarction.

	Group	A	B	C	*p*-Value
Parameter					
Age [years]		64.8 ± 9.8 ^a,b^	65.8 ±.9.8 ^b^	64.4 ± 9.8 ^a^	*p* = 0.0464
Male sex %		65 ^a^	70.5 ^b^	76.1 ^b^	*p* < 0.0001
Stroke %		4.7	3.2	5.2	*p* = 0.1812
TC [mg/dL]		193.7 ± 44.4 ^b^	173.6 ± 49.4 ^a,b^	178.2 ± 43.7 ^a^	*p* = 0.0015
LDL [mg/dL]		123.4 ± 36.2 ^b^	110.3 ± 38.8 ^a,b^	117.7 ± 36.2 ^a^	*p* = 0.0023
HDL [mg/dL]		44.1 ± 14.1	44.3± 11.2	45.2 ± 14.1	*p* = 0.6810
TG [mg/dL]		146.1 ± 95.6	142.3 ± 75.0	131.5 ±95.7	*p* = 0.2200
Creatinine		1.03 ± 0.44	1.13 ± 0.78	1.07 ± 0.5	*p* = 0.180
EF%		45.8 ± 12.3 ^b^	44.9 ± 12.2 ^a,b^	42.1 ± 12.6 ^a^	*p* = 0.0413

^a,b,c^—groups followed by the same letter do not differ significantly at significance level α = 0.05. Abbreviations: TC—total cholesterol, LDL—low-density lipoprotein, HDL—high-density lipoprotein, TG—triglycerides, EF—ejection fraction.

**Table 4:** Results of one-dimensional logistic regression for subgroups A vs. B + C (B + C as a reference).

Parameter	OR	95% CI
Number of procedures (3 and more)	1.32	[1.13; 1.54]
Restenosis	0.26	[0.18; 0.37]
Male sex	0.65	[0.56; 0.76]
NSTEMI	2.76	[1.91; 3.99]
AH	0.51	[0.38; 0.67]
DM	0.79	[0.64; 0.97]
Smoking habit	1.36	[1.01; 1.82]
Occlusion in LAD	0.73	[1.51; 1.98]
Occlusion in D	1.66	[1.31; 2.12]
Occlusion in RCA	1.39	[1.21; 1.59]
Number of critically narrowed arteries		
1	1	-
2	1.6	[1.36; 1.88]
3	2.04	[1.59; 2.61]
Thrombectomy	7.56	[3.92; 14.57]
BMS	2.15	[1.88; 2.48]
POBA	0.39	[0.29; 0.52]
Direct stenting	1.16	[1.02; 1.34]
Sinus rhythm	0.72	[0.55; 0.93]
AF	1.47	[1.09; 1.99]
ST elevation	2.35	[1.85; 2.99]
ST depression	1.46	[1.22; 1.76]
Negative T wave	0.83	[0.71; 0.97]
Absence of R progression	0.73	[0.57; 0.94]
Troponin > upper limit of normal (ULN)	1.75	[1.37; 2.24]
Troponin 3x > ULN	1.73	[1.34; 2.22]
Troponin 5x > ULN	1.63	[1.26; 2.09]
TC	1.008	[1.003; 1.013]
LDL	1.009	[1.003; 1.015]
EF	1.02	[1.01; 1.04]

Abbreviations: AF—atrial fibrillation, BMS—bare metal stent, D—diagonal, DM—type 2 diabetes, EF—ejection fraction, AH—arterial hypertension, LAD—left anterior descending, LDL—low-density lipoprotein, NSTEMI—non-ST elevation myocardial infarction, POBA—plain old balloon angioplasty, RCA—right coronary artery, TC—total cholesterol.

## Data Availability

The data presented in this study are available on request from the corresponding author.

## References

[1] Knuuti J., Wijns W., Saraste A., Capodanno D., Barbato E., Funck-Brentano C., Prescott E., Storey R.F., Deaton C., Cuisset T. (2020). ESC Scientific Document Group, 2019 ESC Guidelines for the diagnosis and management of chronic coronary syndromes: The Task Force for the diagnosis and management of chronic coronary syndromes of the European Society of Cardiology (ESC). Eur. Heart J..

[2] Hiczkiewicz J., Burchardt P., Pieszko K., Budzianowski J., Hiczkiewicz D., Musielak B., Winnicka-Zielińska A., Adamczak D., Faron W., Rzeźniczak J. (2020). The risk for subsequent coronary interventions in a local Polish population. Adv. Interv. Cardiol..

[3] Ford T.J., Berry C. (2019). How to Diagnose and Manage Angina Without Obstructive Coronary Artery Disease: Lessons from the British Heart Foundation CorMicA Trial. Interv. Cardiol..

[4] Cook S., Walker A., Hugli O., Togni M., Meier B. (2007). Percutaneous coronary interventions in Europe. Clin. Res. Cardiol..

[5] Patel M.R., Peterson E.D., Dai D., Brennan J.M., Redberg R.F., Anderson H.V., Brindis R.G., Douglas P.S. (2010). Low diagnostic yield of elective coronary angiography. N. Engl. J. Med..

[6] Ford T.J., Corcoran D., Sidik N., Rocchiccioli P., McEntegart M., Berry C. (2019). MINOCA: Requirement for definitive diagnostic work-up. Heart Lung Circ..

[7] Stone G.W., Hochman J.S., Williams D.O., Boden W.E., Ferguson T.B., Harrington R.A., Maron D.J. (2016). Medical Therapy With Versus Without Revascularization in Stable Patients With Moderate and Severe Ischemia. J. Am. Coll. Cardiol..

[8] Rajkumar C.A., Nijjer S.S., Cole G.D., Al-Lamee R., Francis D.P. (2018). “Faith Healing” and “Subtraction Anxiety” in Unblinded Trials of Procedures. Circ. Cardiovasc. Qual. Outcomes.

[9] Stone G.W., Maehara A., Lansky A.J., De Bruyne B., Cristea E., Mintz G.S., Mehran R., McPherson J., Farhat N., Marso S.P. (2011). A prospective natural-history study of coronary atherosclerosis. N. Engl. J. Med..

[10] Ahmadi A., Leipsic J., Blankstein R., Taylor C., Hecht H., Stone G.W., Narula J. (2015). Do plaques rapidly progress prior to myocardial infarction? The interplay between plaque vulnerability and progression. Circ. Res..

[11] Fernández-Friera L., Fuster V., López-Melgar B., Oliva B., García-Ruiz J.M., Mendiguren J., Bueno H., Pocock S., Ibanez B., Fernández-Ortiz A. (2017). Normal LDL-Cholesterol Levels Are Associated With Subclinical Atherosclerosis in the Absence of Risk Factors. J. Am. Coll. Cardiol..

[12] Nambi V., Bhatt D.L. (2017). Primary Prevention of Atherosclerosis: Time to Take a Selfie?. J. Am. Coll. Cardiol..

[13] Gerber Y., Weston S.A., Enriquez-Sarano M., Manemann S.M., Chamberlain A.M., Jiang R., Roger V.L. (2016). Atherosclerotic Burden and Heart Failure After Myocardial Infarction. JAMA Cardiol..

[14] Maddox T.M., Ho P.M., Roe M., Dai D., Tsai T.T., Rumsfeld J.S. (2010). Utilization of secondary prevention therapies in patients with nonobstructive coronary artery disease identified during cardiac catheterization: Insights from the National Cardiovascular Data Registry Cath-PCI Registry. Circ. Cardiovasc. Qual. Outcomes.

[15] Dwyer J.P., Redfern J., Freedman S.B. (2008). Low utilisation of cardiovascular risk reducing therapy in patients with acute coronary syndromes and non-obstructive coronary artery disease. Int. J. Cardiol..

[16] Libby P., Theroux P. (2005). Pathophysiology of coronary artery disease. Circulation.

[17] Little W.C., Constantinescu M., Applegate R.J., Kutcher M.A., Burrows M.T., Kahl F.R., Santamore W.P. (1988). Can coronary angiography predict the site of a subsequent myocardial infarction in patients with mild-to-moderate coronary artery disease?. Circulation.

[18] Maddox T.M., Stanislawski M.A., Grunwald G.K., Bradley S.M., Ho P.M., Tsai T.T., Rumsfeld J.S. (2014). Nonobstructive coronary artery disease and risk of myocardial infarction. JAMA.

[19] Kotseva K., De Backer G., De Bacquer D., Rydén L., Hoes A., Grobbee D., Maggioni A., Marques-Vidal P., Jennings C., Abreu A. (2019). EUROASPIRE Investigators*. Lifestyle and impact on cardiovascular risk factor control in coronary patients across 27 countries: Results from the European Society of Cardiology ESC-EORP EUROASPIRE V registry. Eur. J. Prev. Cardiol..

